# Automatic and Direct Identification of Blink Components from Scalp EEG

**DOI:** 10.3390/s130810783

**Published:** 2013-08-16

**Authors:** Wanzeng Kong, Zhanpeng Zhou, Sanqing Hu, Jianhai Zhang, Fabio Babiloni, Guojun Dai

**Affiliations:** 1 College of Computer Science, Hangzhou Dianzi University, Hangzhou 310018, China; E-Mails: 110061062@hdu.edu.cn (Z.Z.); sqhu@hdu.edu.cn (S.H.); jhzhang@hdu.edu.cn (J.Z.); daigj@hdu.edu.cn (G.D.); 2 Department of Biomedical Engineering, University of Minnesota, Minneapolis, MN 55455, USA; 3 Department of Physiology and Pharmacology, University of Rome “Sapienza”, Rome 00185, Italy; E-Mail: fabio.babiloni@uniroma1.it; 4 IRCCS Fondazione Santa Lucia, via Ardeatina 306, Rome 00179, Italy

**Keywords:** scalp EEG, correlation, eye blink artifact, independent component analysis (ICA), identify

## Abstract

Eye blink is an important and inevitable artifact during scalp electroencephalogram (EEG) recording. The main problem in EEG signal processing is how to identify eye blink components automatically with independent component analysis (ICA). Taking into account the fact that the eye blink as an external source has a higher sum of correlation with frontal EEG channels than all other sources due to both its location and significant amplitude, in this paper, we proposed a method based on correlation index and the feature of power distribution to automatically detect eye blink components. Furthermore, we prove mathematically that the correlation between independent components and scalp EEG channels can be translating directly from the mixing matrix of ICA. This helps to simplify calculations and understand the implications of the correlation. The proposed method doesn't need to select a template or thresholds in advance, and it works without simultaneously recording an electrooculography (EOG) reference. The experimental results demonstrate that the proposed method can automatically recognize eye blink components with a high accuracy on entire datasets from 15 subjects.

## 1. Introduction

Eye blink is inevitable during long-term scalp electroencephalogram (EEG) recording and it is an important artifact component for removal or reuse during EEG analysis. Regarding its removal, because of the high amplitude of eye blink, it has a significant effect on the results of EEG analysis [1–3]. Especially for event-related-potential (ERP) experiments, the eye blink will disturb the evoked potential, and lead to decision mistakes [4–6]. Regarding its reuse, the rate of eye blink can be used to assess mental states like drowsiness [7–11]. Hence, it is important to separate and identify the eye blink components from EEG signals for the purpose of artifact removal or reuse in special applications.

There are numerous methods to separate ocular artifacts (OA) from EEG signals either in the time domain or frequency domain [12–19]. One the most popular categories of methods is based on blind source separation (BSS) [12,13]. It has been proved that ICA is an effective and generally applicable method for treating artifacts, including OA separation [14,15]. However, conventional artifact correction based on ICA relies on manual inspection of Independent Components (ICs) to decide which ICs are eye blink artifacts, so they are time consuming and can't be used in many automatic online applications. Hence, it is essential to develop a system which can identify the blink ICs with an automatic manner.

In recent years, many researchers have worked in this field and achieved some significant results [17–26]. Joyce *et al.* [17] used Second Order Blind Identification (SOBI) algorithm along with correlation metrics with electrooculography (EOG) reference channels for automatic removal of artifacts. This is the first time the ICA-based method was employed for automatic OA removal. However, the method needs EOG reference channels which is not convenient for subjects and may not be suitable for some cases without EOG recording. Subsequently, Ning-Yan Bian, *et al.* [18] analyzed each IC by three exponents: Largest Lyapunov Exponent, Kurtosis and Hurst Exponent, to identify the artifact components, such as eye blinks and muscle movement. Herrero [20] employed fractal dimension (FD) which was a measure of signal complexity, to distinguish ocular and neural activity. Mammone [21] proposed a technique based on ICA and Renyi's entropy to automatically detect artifacts including eye blinks. It was more effective than another ICA-based approach which jointly used kurtosis and Shannon's entropy [22]. In [23], sample entropy, a measure of data regularity, was used to identify the eye blink artifact component. However, almost all the aforementioned methods need to set a rejection threshold to confirm blink components, in other words, they are not fully automatic. Furthermore, the above outlined research only focused on the case of EEG epochs with blinks, and none had been tested on the entire dataset which may contain epochs without blinks. In addition, some methods are aimed at removing various artifacts, so it is difficult to find appropriate thresholds for different artifacts. Furthermore, some non-physiological artifacts, for example, baseline drift, may affect the performance of the detection.

There are also some methods which are based on the difference of scalp topography between eye blink component and neural components. Delsanto *et al.* [24] defined a model of the topographic maps associated with the eye blink component and a measure of the resemblance between different components was used for automatic identification of eye blink artifact. In [25], a new method was presented, which used the scalp topography pattern of blink IC as a template and selected the component in other epochs whose scalp topography is the most similar with the template as blink IC. Phothisonothai *et al.* [26] jointly used normalized weight matrix and frontal head regions to identify blink components, and they also integrated other indexes such as kurtosis, fractal dimension and so on to confirm other artifacts. This method introduced spatial information to assist identifying blink components. However, it had to work with thresholds. Finally, Mognon *et al.* [27] developed a completely automatic algorithm that identified artifact components by combining spatial and temporal features. Kurtosis and spatial average difference between different areas (scalp areas were divided into four parts, *i.e.*, Frontal, Posterior, Left Eye and Right Eye) were calculated to determine the eye blink artifact. It is quite a good method to recognize artifacts. The main limitation of the algorithm is that it requires electrodes distributing on the whole scalp. However, some experiments' montages don't meet the requirement, and so it may not work in certain cases.

In addition to ICA-based methods, regression methods also play important roles in removal of EOG artifacts including blinks. The most successful approach was proposed by A. Schlögl *et al*. [16]. It is also a fully automated method for removing EOG artifacts. The EEG can be corrected by subtracting weighted noise (*i.e.*, EOG) from signals, while weight coefficients can be calculated fast by regression from signals (*i.e.*, EEG) and noise (*i.e.*, EOG). The only downside of this method is that it needs separated EOG channels recording EOG simultaneously.

In this paper both the correlation between independent components and frontal EEG signals and attributions of mixing matrix of ICA are investigated. Then a fully automatic method based correlation index and the feature of power distribution is proposed for recognizing eye blink components. The proposed method has five advantages: (1) it is free from thresholds, so that it is smart enough to cope with EEG signals from various subjects; (2) it is free from EOG recording channels, and it is more convenient for subjects; (3) it doesn't need to acquire any templates beforehand; (4) it can detect blink ICs on entire datasets which contain epochs with or without blinks, and (5) it introduces little extra computation, as the proposed index can be simply calculated directly from the mixing matrix ***A*** of ICA. The method was validated by two different EEG complete datasets. Results of experiments show that it is effective to recognize eye blink artifact source automatically from independent components with high accuracy on 15 subjects.

## 2. Method

### 2.1. Independent Component Analysis

ICA is a statistical technique which can separate mixed signals, including EEG signals, to maximally isolate ICs by a specified measure of statistical independence [28,29]. Typically, the ICA problem can be described as below: Let *X* = [*x*_1_, *x*_2_,…,*x_N_*]*^T^* be *N* observed signals from individual scalp EEG channels, and *S* = [*s*_1_, *s*_2_,…,*s_M_*]*^T^* be *M* original unobserved sources. The ICA model can be expressed in the general case as:

(1) X=AS

where ***A*** is a full-rank scalar matrix as [*N* × *M*] that mixes ICs back to observed signals. Since we don't know the effective number of statistically-independent signals contributing to the scalp EEG, according to [14], we can assume that the number of sources is equal to the number of sensors, *i.e.*, *N* = *M*. Given the EEG data, an ICA algorithm can produced the mixing matrix ***A*** and *N* independent sources. The matrix ***A*** is expressed as below:

(2) $$A=\left(\begin{array}{ccc} a_{11} & a_{12}\cdots & a_{1N}\\ \begin{array}{l} a_{21}\\ \cdots \end{array} & \begin{array}{l} a_{22}\cdots \\ \cdots \end{array} & \begin{array}{l} a_{2N}\\ \cdots \end{array}\\ a_{N1} & a_{N2}\cdots & a_{NN} \end{array}\right)$$

where *a_ij_* (1 ≤ *i*, *j* ≤ *N*) is the transfer coefficient from the *j*-th source to the *i*-th observed channel signal. Therefore, the column vector of ***A*** reflects the power propagating across on all scalp channels [30] for one component. And the aim of ICA is to find out the linear unmixing matrix ***W*** and acquire the ICs under the conditions of independent criterions which is an inverse problem of Equation (1), so that:

(3) S=WX

In this paper, the extended infomax ICA was utilized to decompose EEG signals. The reason is that it can take both the sub-Gaussian and sup-Gaussian signals into account [31]. Furthermore, it is quite good at separating eye blink artifact from weaker electrical signals that arise from cerebral cortex.

### 2.2. Premise

According to Equations (1) and (2), we also can get the expression of observed EEG signal for each channel ***x****_i_* as follows:

(4) $$x_{i}=a_{i1}\;s_{1}+a_{i2}\;s_{2}+\cdots +a_{iN}\;s_{N}=\sum_{j=1}^{N}a_{ij}\;s_{j}$$

It is clear that each observed scalp EEG signal is a linear combination with all independent sources. The time courses of the sources from ICA algorithm are supposed independent, since external sources such as eye and muscle activity, heart beating, and device noise are basically not time locked to the internal sources of brain activity decoded by clean EEG signals [14]. Therefore, in this paper, we divided the sources into two parts, *i.e.*, internal sources and external sources, considering that the internal sources are cerebral sources that we are interested in, while external sources are artifacts consisting of eye blinks, head movement, muscle activity, cardiac signals and so on. Accordingly, the observed EEG signal for each channel can be expressed as a linear combination of two kinds of sources as follows:

(5) $$x_{i}=\underset{\begin{array}{cc} \text{internal} & \text{sources} \end{array}}{\underset{︸}{a_{i1}\;s_{1}+a_{i2}\;s_{2}+\cdots +a_{ik}\;s_{k}}}+\underset{\begin{array}{cc} \text{external} & \text{sources} \end{array}}{\underset{︸}{a_{i(k+1)}\;s_{k+1}+\cdots +a_{iN}\;s_{N}}}$$

Eye blink causes a change in the electric fields that surround the eyes, which in turn affects the electric field over the frontal scalp generated by neural potentials. The propagation of power of eye blink to scalp mainly depends on the filter properties of the subject's scalp, skull and neuronal tissues. Given the high resistivity of the skull, the scalp is a major conductive medium for eye blink [6]. For the most part, the conductive properties of the scalp skin are relatively constant over time but proportional over distance. Hence, the eye blink affects the scalp EEG channels to different extents corresponding to geodesic distance between EEG channel locations and eyes. Especially, the frontal scalp channels are impacted most by eye blinks. For cerebral signals, *i.e.*, internal sources, they have to pass through the fluids surrounding the brain, then the skull, and finally the scalp, therefore, both the amplitude and the power will be evidently decayed as they propagate until they're detected by electrodes. We can deduce a conclusion from the premise that the eye blink artifact has a more significant impact on the temporal correlation with frontal scalp signals than internal cerebral activity. Furthermore, the style of the power topography of eye blink component is just like ripple diffusion when a stone is threw into water. And the eye blink is corresponding to the source of stone. More detail, the power on scalp is gradually decayed as the distance increases from the source of eye blink namely the center of the brow. Here, we proposed a fully automatic and direct method that utilizes both temporal correlation and power distribution to detect eye blink components with high accuracy.

### 2.3. The Correlation Based Index Method

In this paper, the Pearson product-moment correlation coefficient (PCC) was employed to show the strength of correlation between the component and the observed EEG signal. The PCC is a statistical measure of the linear correlation or dependence between two time-series variables *X* and *Y*, and its value ranges from −1 to 1 [32].The greater the correlation coefficient, the higher degree of the linear relationship is. The PCC formula is as follows:

(6) $$\rho (X,Y)=\frac{\text{Cov}(X,Y)}{\sqrt{D(X)}\sqrt{D(Y)}}=\frac{E\left\{\left[X-E(X)][Y-E(Y)\right]\right\}}{\sqrt{D(X)}\sqrt{D(Y)}}$$

In order to distinguish eye blink component from other components, the sum of correlation is calculated against frontal scalp EEG signals for each component. And we refer to this quality as “Correlation based Index” (CBI). The formula is shown below:

(7) $$\text{CBI}(j)=\sum_{i=1}^{k}\left|\rho (x_{i},s_{j})\right|1\leq j\leq N,x_{i}\in \text{frontal electrodes},1\leq i\leq k$$

What does the CBI index mean actually? Now, we try to illustrate it with a totally novel perspective of power distribution by using the transfer coefficient *a_ij_*.

First, if variable *X* and *Y* are mutual independence, so:

(8) E{[X−E(X)][Y−E(Y)]}=0

where *E* is the expectation operator. According to Equation (4), we can deduce the correlation between one channel of scalp EEG signal and one certain independent component source as follows:

(9) $$\begin{array}{ll} \rho (x_{i},s_{j}) & =\frac{E\left\{\left[x_{i}-E(x_{i})][s_{j}-E(s_{j})\right]\right\}}{\sqrt{D(x_{i})}\sqrt{D(s_{j})}}\\ & =\frac{E\left\{\left[a_{i1}s_{1}(t)+a_{i2}s_{2}(t)+\cdots +a_{iN}s_{N}(t)-E(a_{i1}s_{1}(t)+a_{i2}s_{2}(t)+\cdots +a_{iN}s_{N}(t))][s_{j}-E(s_{j})\right]\right\}}{\sqrt{D(x_{i})}\sqrt{D(s_{j})}}\\ & =\frac{E\left\{\left[a_{i1}(s_{1}-E(s_{1}))+a_{i2}(s_{2}-E(s_{2}))+\cdots +a_{iN}(s_{N}-E(s_{N}))][s_{j}-E(s_{j})\right]\right\}}{\sqrt{D(x_{i})}\sqrt{D(s_{j})}} \end{array}$$

Because components are independent, so we can utilize Equation (8) and obtain:

(10) $$\rho (x_{i},s_{j})=\frac{E\left\{\left[a_{ij}(s_{j}-E(s_{j}))][s_{j}-E(s_{j})\right]\right\}}{\sqrt{D(x_{i})}\sqrt{D(s_{j})}}=\frac{a_{ij}D(s_{j})}{\sqrt{D(x_{i})}\sqrt{D(s_{j})}}$$

Furthermore, let Equation (4) substitute into the function of *D*(*x_i_*) to eliminate the term of *x_i_*. *D*(*x_i_*) can be simplified as follows:

(11) $$\begin{array}{ll} D(x_{i}) & =E\{{[a_{i1}s_{1}(t)+a_{i2}s_{2}(t)+\cdots +a_{iN}s_{N}(t)-E(a_{i1}s_{1}(t)+a_{i2}s_{2}(t)+\cdots +a_{iN}s_{N}(t))]}^{2}\}\\ & =E\{{\left[a_{i1}\text{(}s_{1}-E(s_{1}))+a_{i2}\text{(}s_{2}-E(s_{2}))+\cdots +a_{iN}\text{(}s_{N}-E(s_{N}))\right]}^{2}\}\\ & =a_{i1}^{2}\text{E}\{{\left[s_{1}-Es_{1}\right]}^{2}\}+a_{i2}^{2}\text{E}\{{\left[s_{2}-Es_{2}\right]}^{2}\}+\cdots +a_{iN}^{2}\text{E}\{{\left[s_{N}-Es_{N}\right]}^{2}\}\\ & =a_{i1}^{2}D(s_{1})+a_{i2}^{2}D(s_{2})+\cdots +a_{iN}^{2}D(s_{N}) \end{array}$$

According to Equations (10) and (11), we can deduce the correlation between one channel of scalp EEG and one independent component with a much more intuitive form:

(12) $$\rho (x_{i},s_{j})=\frac{a_{ij}\sqrt{D(s_{j})}}{\sqrt{a_{i1}^{2}D(s_{1})+a_{i2}^{2}D(s_{2})+\cdots +a_{iN}^{2}D(s_{N})}}$$

In order to further simplify Equation (12), the variance of each component can be normalized as *D*(*s_j_*) = 1 (1 ≤ *j* ≤ *N*) after ICA. Actually, in many ICA algorithms, the variance of the independent component is satisfied with *D*(*s_j_*) = 1 [30]. After that, we have the simpler expression for Equation (12) as follows:

(13) $$\rho (x_{i},s_{j})=\frac{a_{ij}}{\sqrt{a_{i1}^{2}+a_{i2}^{2}+\cdots +a_{iN}^{2}}}$$

Equation (13) indicates that the correlation between the *j*-th source and the *i*-th channel of scalp EEG signal is equivalent to the normalized proportion of the transfer coefficient *a_ij_* to the root mean square (RMS) of transfer coefficients for all sources on the *i*-th channel's signal. In view of Equation (13), we can conclude that the stronger the correlation, the more the power propagating from the component to the scalp channel will be.

So, finally, we can deduce the correlation based index (CBI) as follows:

(14) $$\text{CBI}(j)=\sum_{i=1}^{k}\left|\rho (x_{i},s_{j})\right|=\sum_{i=1}^{k}\frac{\left|a_{ij}\right|}{\sqrt{a_{i1}^{2}+a_{i2}^{2}+\cdots +a_{iN}^{2}}}\;x_{i}\in \text{frontal electrodes},1\leq i\leq k$$

**Figure 1.**
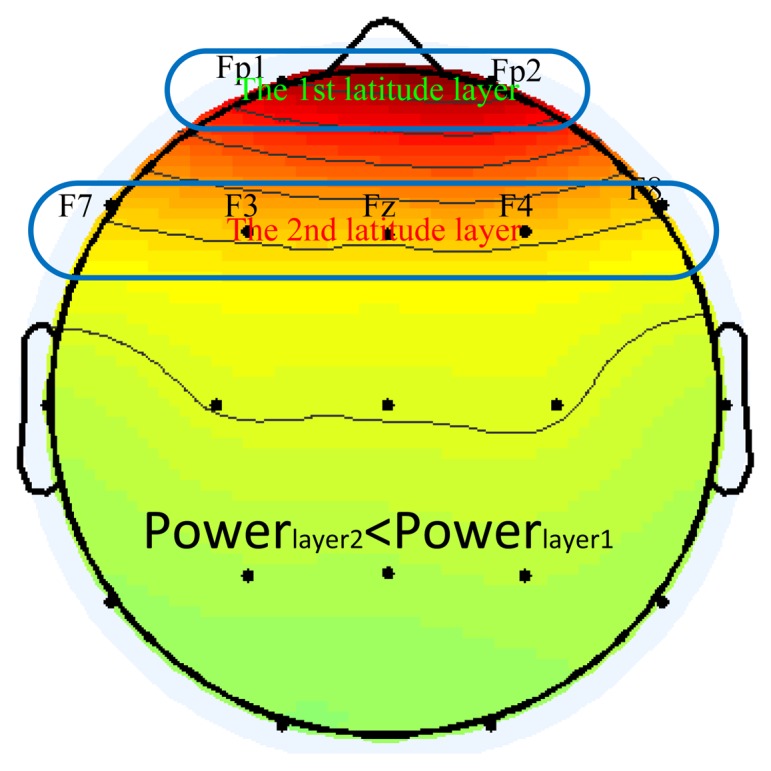
Power topographic of the blink components and two different layers according to different latitude line. The power in the second layer must be less than the one in first layer.

According to our premise that the frontal scalp channels are impacted most by eye blinks, the CBI value of blink component must be the largest one if the EEG epoch contains eye blinks. Hence, we can calculate the CBI value for each component, then, find the component with largest CBI value to be initially identified as eye blink. However, in the entire dataset, it is unavoidable to encounter the problem that there are no blink components at all because the epoch itself is without blinks. In that case, we should employ the power distribution at the front scalp as a supplementary mean to assist to detect blink components accurately. In order to judge the candidate component is blink or not with power topographic, we partition frontal scalp channels into different layers according to different latitudes as Figure 1. Actually, EEG montage is always built up according to international 10–20 system, thus, we can easily range channels on the same line of latitude into the same layer. We also define that the nearest layer from nose is the first layer and other layers are listed in order of radial distance from the nose. If the *j*-th component is blink, its power distribution must satisfy the rule that any channel's power in first layer is stronger than anyone in the second layer. Specifically, that is *a_ij_* > *a_kj_* ∀*i* ∈ the 1st latitude layer, *k* ∈ the 2nd latitude layer. Of course, in order to increase the detection accuracy of blink components, the number of layers in the frontal scalp for the rule can be increased to 3 if it is necessary.

### 2.4. Summary of the Proposed Method

In this paper, we proposed a totally automatic method which combined correlation index with power distribution to identify the blink artifact component. The method involves the following steps:

(1) ICA for the EEG data, produce *N* independent components and mixing matrix ***A***;
(2) Calculate the *CBI*(*j*) for each component according to Equation (14);
(3) Find the component which has the largest value of CBI and regard it as the blink candidate component.
(4) Confirm the candidate component with the power distribution condition as *a_ij_* > *a_kj_* ∀*i* ∈ 1st latitude layer *k* ∈ the 2nd latitude layer. If the candidate component satisfies the above condition, then it is eye blink component, else, we can judge that the analyzed epoch is no blinks.
(5) After the eye blink artifact component is determined, we can set the column of ***A*** corresponding to the eye blink artifact IC to zero and reconstruct the signals according to Equation (1) for artifact removal or analyze separately with the eye blink component for reuse in special applications.

**Figure 2.**
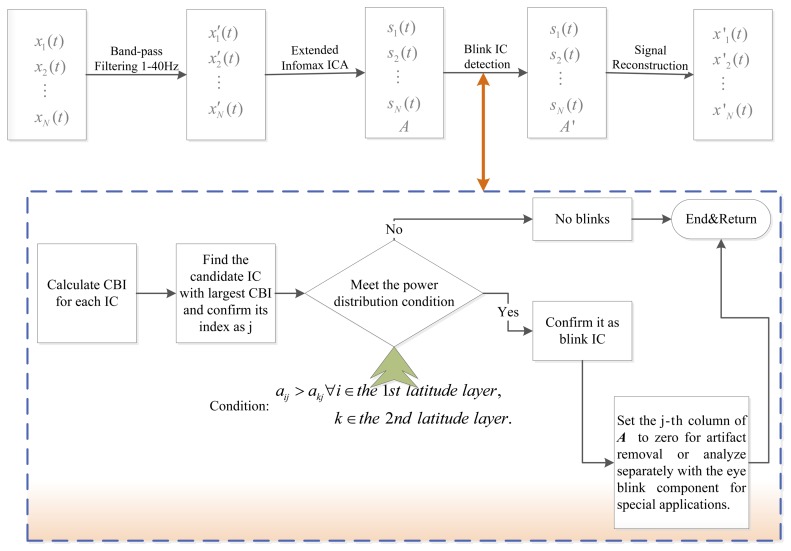
Principle diagram of the proposed method.

The principle diagram of the method is given in Figure 2.

It is worth mentioning that our method is a really full-automatic approach to identify the eye blink without any thresholds and EOG reference channels. Furthermore, it is very simple to compute the feature index as Equation (14) after ICA, for it is transformed directly from mixing matrix ***A***. Hence we actually needn't calculate the Pearson product-moment correlation coefficient (PCC) for CBI.

## 3. Experimental Results

### 3.1. Data Acquisition

Two different EEG datasets were used in this paper. One dataset was recorded with a 1,000 Hz sampling rate using a Neuroscan 64-channel system aiming at collecting EEG signals contaminated by eye blinks. Subjects are required to blink eyes about once every 2–3 s. Specifically, 62 channels were used to record scalp EEG and the other two were used to record vertical electrooculogram (VEOG) and horizontal electrooculogram (HEOG). Right earlobe was used as reference. During the EEG recording, subjects were seated on a chair without performing tasks, and the recording lasted 30 min. The dataset was collected from five subjects.

The second dataset was collected from 10 volunteers for the neuromarketing experiments, and subjects are all mental healthy with five males and five females aged from 22 to 25 years [33]. As it was a normal scalp EEG recording experiment, the frequency of eye blink is much lower than the first dataset. In other words, there will be many more no-blinks epochs than in the first dataset. Subjects were comfortably seated on an armchair in an isolated and quiet room, and they watched a video which contains both a neutral documentary of 8 min and six TV commercials of 3 min duration without knowing the purpose of the experiment. EEG data was collected with a 256-HZ sampling rate using a g.Tec 16-channel system while the impedances were kept below 5 kΩ. The electrode placement was built according to the international 10–20 system. Specifically, 15 electrodes are used to record EEG activity and they are AF3, Af4, F3, F4, FPz, Fz, Cz, C3, C4, Pz, POz, T7, T8, P3, and P4. The rest one channel is used to record EKG signal. Right earlobe was used as reference. All signals were band-pass filtered with a cutoff frequency of 1–40 Hz.

### 3.2. Validation of the Proposed Method

To ensure a good separation performance of the ICA algorithm, as a general rule, finding *N* stable components typically requires more than *k* × *N*^2^ data sample points at each channel [34], where *k* is a multiplier about 30, if *N* ≤ 32. For this reason, the segment length of epoch was set to be 10 s.

**Figure 3.**
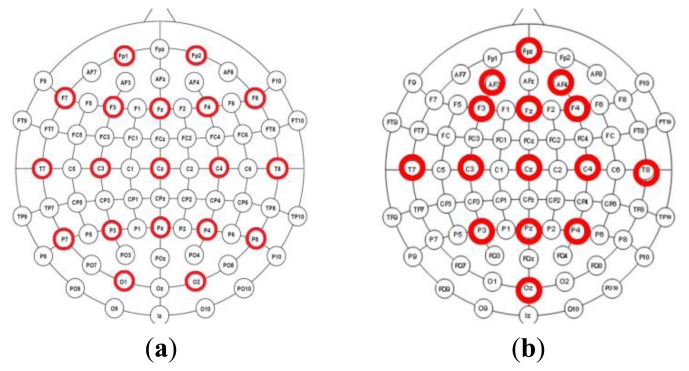
(**a**) The montage in the first dataset is responding to condition 1; (**b**) The montage of the second dataset from the neuromarketing experiment is responding to condition 2.

Two datasets are divided into two different montage conditions. For the first condition, 19 channels EEG data were selected, they are Fp1, Fp2, F7, F3, Fz, F4, F8, T7, C3, Cz, C4, T8, P7, P3, Pz, P4, P8, O1, O2 and distributed over the entire scalp in the first dataset. For the second condition, we selected 15 channels from the second dataset except EKG. The montages of two conditions are showed as Figure 3.

**Figure 4.**
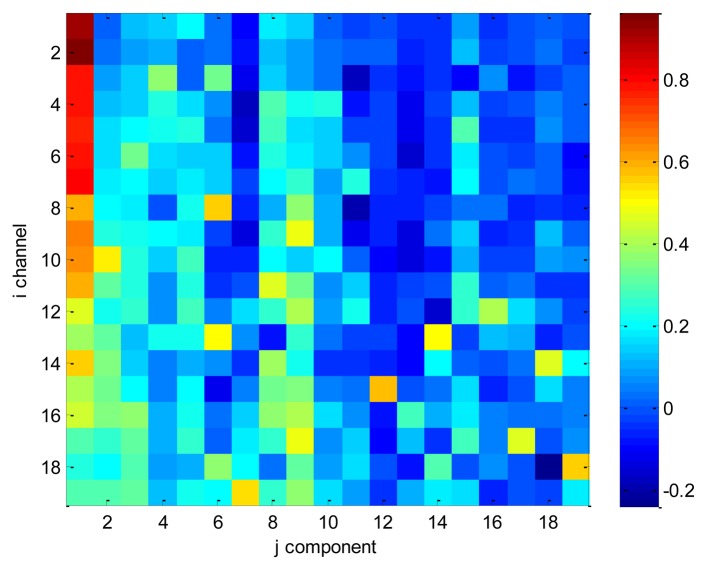
The scatter diagram of correlation value *ρ*(*x_i_*, *s_j_*). The first component is blink IC.

In order to illustrate the CBI index, we selected an epoch of EEG with 19 channels which contained eye blinks, and the montage is referring to Figure 3a. We applied ICA method on the selected data and computed the mixing matrix ***A***. Finally, we scattered the correlation value *ρ*(*x_i_*, *s_j_*) over Figure 4. Independent components have been manually checked and we ascertain that the first component is the eye blink. From Figure 4, we also can conclude that the sum of first column is much larger than other columns. Especially, values of *ρ*(*x*_1_, *s*_1_), and *ρ*(*x*_2_, *s*_1_) are extremely large among all values. Why? According to Figure 3a, we know that channel 1 and 2 are corresponding to Fp1 and Fp2 which are two nearest channels from eyes. It also proves that the CBI index proposed by our paper makes sense for detecting the eye blink components.

**Figure 5.**
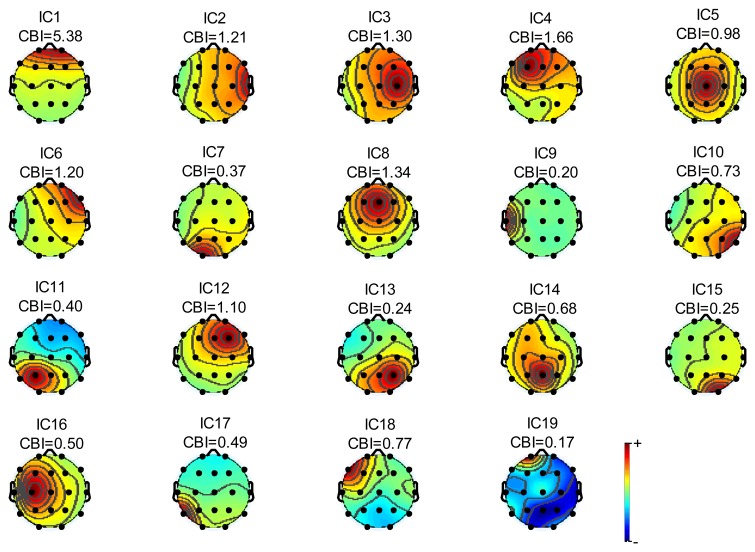
The power topographic of independent components for first condition.

In order to have a clearer outline of our method which use correlation index to distinguish eye blink components, we random selected an epoch of EEG signal which contains eye blinks for analysis. Figure 5 shows power topographies corresponding to different independent components.

The component index is denoted as IC*x* while CBI is the sum of correlation between one component and frontal scalp EEG signals as defined in Equation (14). From Figure 5, we can find that the extremely large CBI happened in IC1. According to our manual matching, IC1 is the eye blink component which is with the largest CBI value. On the other hand, it also illustrates that the CBI value is a significant indicator for distinguishing blink components. Furthermore, the topography feature for IC1 also demonstrates that power decreases gradually as the geodesic distance between EEG channel locations and eyes increases. Meanwhile, the powers of both two electrodes in the first layer are greater than powers of all five electrodes in the second layer. Thus it satisfied the condition of power distribution proposed in this paper. Based on the outline of other topographies, it is easy to recognize IC3, IC4, IC6, IC7, IC8, IC12, IC13, IC14 and IC15 as internal sources.

**Figure 6.**
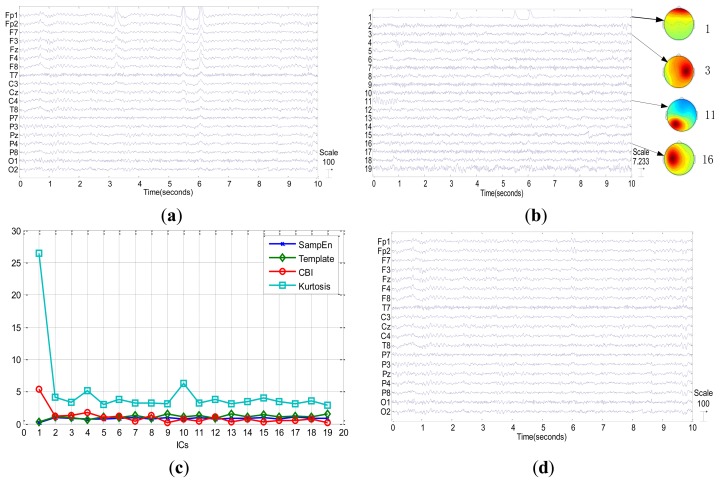
Blinks case. (**a**) Original signals; (**b**) Independent components decomposed by Extended Infomax ICA and power topographies corresponding to some components; (**c**) Values of CBI, sample entropy, Kurtosis and template angle corresponding to all components; (**d**) Reconstructed signals after blink artifact removal by the proposed method.

In this section, we show two examples which contain blinks and no-blinks respectively to illustrate the procedure of the proposed method. Figure 6a shows an example of one epoch original EEG signal with three eye blinks at around 3.2 s, 5.5 s and 6 s, respectively. The data is collected from the first dataset with condition 1. In Figure 6c, the first component has the highest value of CBI, hence, it is the candidate component of eye blink. In order to further confirm it as blink IC, we check its power topography in contrast to condition of power distribution described in Figure 1.

**Figure 7.**
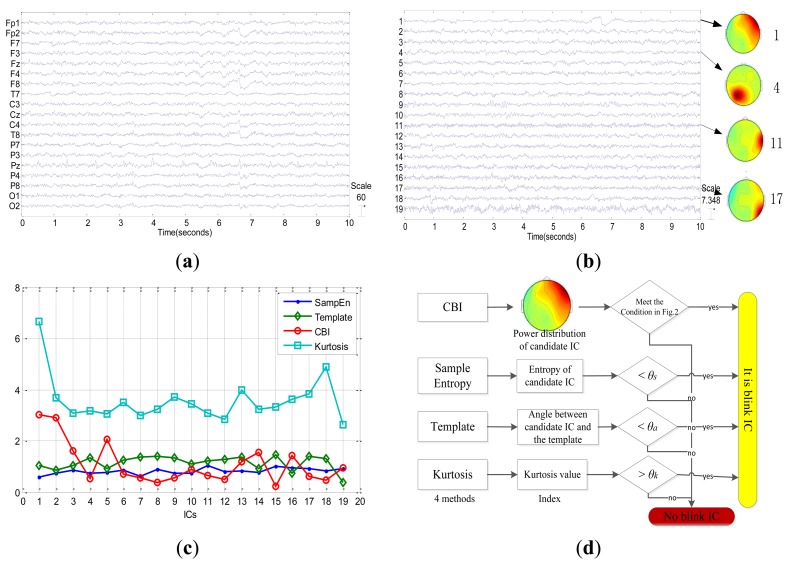
No-blinks case. (**a**) Original signals; (**b**) Independent components decomposed by Extended Infomax ICA and scalp topographies corresponding to weights of some components; (**c**) Values of CBI, sample entropy, kurtosis and template angle corresponding to all components; (**d**) Procedures of judging blink IC for 4 methods.

From Figure 6b, we can find that the power of channels in first layer is stronger than anyone in the second layer for the first component. As a result, we should select the first component as eye blink component according to our method summarized in Figure 1. The identification result of our method is consistent with the result that we have manually checked. Figure 7 shows the other example of EEG signals without any blinks. Although the first component has the highest CBI value as shown in Figure 7c, its power topography as shown in Figure 7b doesn't meet the condition of power distribution. Consequently, we judge that there are no blinks in this epoch of EEG. Four different indexes such as sample entropy, template angle, CBI and kurtosis value are presented in both Figure 6c and Figure 7c. For the case in Figure 6, *i.e.*, epoch with blinks, it is easy to confirm the blink IC by distinguished characteristics which are largest CBI and kurtosis value and smallest sample entropy and template angle. While for the no-blinks case in Figure 7, the other three methods, *i.e.*, kurtosis, template and sample entropy can't confirm blink IC unless they employ thresholds. However, the proposed method only utilized the condition of power distribution to affirm blink IC without any thresholds. Figure 7d shows procedures of judging blink IC for the 4 methods and also demonstrates that the proposed method is free from thresholds. Based on this fact, it is a fully automatic method to detect Blink IC from ICA in real applications. Figure 7c also reveals that components after ICA are not always in descending order with the kurtosis value. In addition, the kurtosis index only takes into account the temporal feature of the component. Therefore, it is not robust to detect blink components. Results of this experiment confirm that the proposed method combining CBI index and condition of power distribution is technically feasible.

After automatically recognizing the eye blink components, we set the column of ***A*** corresponding to the identified eye blink component to be zero for artifact removal, and reconstruct signals according to Equation (1). Results demonstrate that the reconstructed signals can eliminate the eye blink effectively as shown in Figure 6d. It is obvious that eye blinks are corrected removed with the proposed method. Furthermore, the proposed method doesn't need to obtain templates or thresholds in advance. From this perspective, it is a totally self-tuned approach to detecting blink components based on ICA.

### 3.3. Eye Blinks Component Detection

We employed ICA to decompose all epochs of two datasets and recorded all ICs for each epoch, then, the proposed method was used for recognizing the eye blink artifact IC automatically. After that, a manual detection was carried out by an experienced expert. The criteria used to recognize blink artifact IC was in view of both time course and topographic maps of ICs. The independent component detection rate (ICDR) [35] was adopted to quantify the overall performance of the proposed method. Actually, it was defined as a ratio of the number of correctly detected eye blink ICs by a method to the total number of analyzed eye blink ICs. In this section, we compare our method with other three methods on two different datasets.

In the first dataset, subjects were required to blink eyes about once every 2–3 s. Hence, the first dataset includes 153 epochs which are recorded from five different subjects to make sure each epoch contained blinks. Furthermore, we compared our method with other three classical methods which are based on sample entropy [23], blink template [25] and kurtosis index [36], respectively. The purpose of experiment in this dataset is to test the total performance of blink detection for four methods. The experiment result is shown in Table 1. The ICDR of our method is 97.4%, while the ICDR of sample entropy-based method, template-based method and kurtosis index are 94.1%, 96.7% and 75.2% respectively. In this dataset, all epochs contain blinks, hence, it doesn't need to confirm the candidate is eye blink or not further. Practically speaking, we just choose the component with largest CBI and kurtosis value as blink IC for the proposed and kurtosis methods respectively, while choosing the component with smallest entropy or angle as blink IC for the other two methods. Nevertheless, these simple mechanisms are only limited to the case in which all epochs contain blinks. And they will not work in the entire dataset from the practical experiment, as it is often the case in which there are no blinks in the epochs.

Considering the practical case of blink detection, the second dataset is from neuromaketing experiment. The frequency of eye blink is low, therefore, there are more epochs without blinks than in the first dataset. The purpose of this experiment is to evaluate the performance of the proposed method for real online applications. Table 2 shows the comparison result of 4 methods on the second dataset with Condition 2. Although the sample entropy and template-based methods succeed in detecting blink ICs in epochs with blinks, it fails to judge whether the component is a blink or not in the case of epochs without blinks. In that case, we must introduce thresholds for both the sample entropy and template-based methods. However, how to select thresholds is an important and open issue for both methods. Although the threshold will have a significant influence on the detection results, there are no guidelines on how to select it. Hence, we have to try different thresholds to achieve the best results for the sample entropy and template-based methods respectively. Finally, the threshold (denoted as *θ* in Table 2) in the template-based method was selected as 0.3 while in the sample entropy it varied from individual to individual. For the kurtosis method in the real application, we should normalize the kurtosis value and select the threshold as 1.64 according to [36]. By contrast, the proposed method was totally free from thresholds, and it just utilized the CBI value and power distribution of frontal scalp to automatically detect blink components. The result of comparing four methods on the second dataset is shown in Table 2. For the sake of clarity, the numbers of epochs with blinks and without blinks are marked in green and orange respectively. The average accuracy and stand deviation for the 4 methods on 10 subjects were also calculated. From Table 2, we find that the CBI and Template methods can succeed in detecting of blink IC in all epochs, and the performance of sample entropy is not as good as the two methods despite of various thresholds. Moreover, the performance of kurtosis method is the worst among all four methods. The main reason is that the kurtosis index only focuses on the temporal feature of components but neglects the spatial information. Hence it is easy to confuse the blinks with other artifacts. The proposed method takes both the temporal and spatial characteristics of components into account, therefore, the CBI method interacts well with the mechanism of blinks. Although the standard deviation of the template-based method is little less than the one of CBI, it needs to manually select template and try different thresholds in advance. Actually, both the template and sample entropy methods cannot be applied for online practical sessions. In general, the proposed method is much better than the other three methods both in terms of detection performance and automatic mode.

### 3.4. Performance Evaluations

In order to evaluate how clean the corrected EEG is after performing our method, we compare the signal-to-noise ratio (SNR) between previous and later stages for correction of blink artifact. First, we select an epoch of EEG signal with 19 channels from the first dataset which contains several blinks, and we treat it as original EEG. Meanwhile, ICA is run on it to produce various components including blink IC. Then, we utilize the EOG channels and regression approach [16] to have EEG signal corrected, and we treat it as clean EEG. Finally, we reconstruct mixed EEG signals *EEG_mixed_* (*t*) from clean EEG *EEG_clean_* (*t*) and blink independent components as follows:

(15) $$EEG_{\text{mixed}}^{i}(t)=EEG_{\text{clean}}^{i}(t)+\lambda_{i}IC_{\text{blink}}(t)$$

where *i* is the index of channel, *IC_blink_*(*t*) is a blink component manually selected based on independent components decomposition of original EEG, and *λ_i_* is the weight on the *i*-th channel for blinks. Considering power propagation on scalp for eye blink, we should choose reasonable *λ_i_* for each channel. One logical method is to utilize the column of mixing matrix in ICA which is corresponding to blink component. Consequently, we employ the proposed method to detect blink components and correct EEG signal as described in Figure 1. In order to evaluate how clean the corrected EEG is, we introduce signal-to-noise ratio (SNR) in this paper. We defined the SNR before correction of blink artifact as follows:

(16) $$SNR_{\text{before}}=\frac{\sqrt{\frac{1}{K\cdot T}\overset{K}{\underset{i=1}{\text{∑}}}\overset{T}{\underset{t=1}{\text{∑}}}{\left(EEG_{\text{clean}}^{i}(t)\right)}^{2}}}{\sqrt{\frac{1}{K\cdot T}\overset{K}{\underset{i=1}{\text{∑}}}\overset{T}{\underset{t=1}{\text{∑}}}{\left(\lambda_{i}IC_{\text{blink}}(t)\right)}^{2}}}$$

where *K* is the number of recording channels and *T* is the number of samples in an epoch. While the SNR after correction of blink artifact can be defined as follows:

(17) $$SNR_{\text{after}}=\frac{\sqrt{\frac{1}{K\cdot T}\overset{K}{\underset{i=1}{\text{∑}}}\overset{T}{\underset{t=1}{\text{∑}}}{\left(EEG_{\text{clean}}^{i}(t)\right)}^{2}}}{\sqrt{\frac{1}{K\cdot T}\overset{K}{\underset{i=1}{\text{∑}}}\overset{T}{\underset{t=1}{\text{∑}}}{\left(EEG_{\text{clean}}^{i}(t)-EEG_{\text{corrected}}^{i}(t)\right)}^{2}}}$$

**Figure 8.**
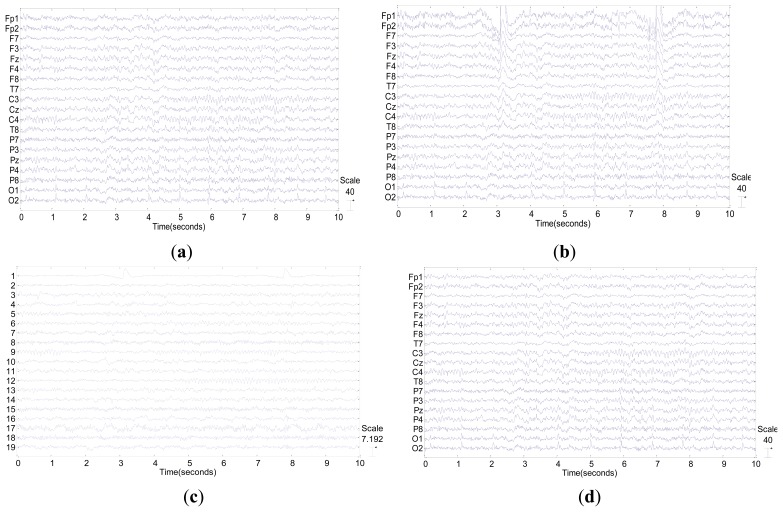
(**a**) An epoch of clean EEG with 19 channels; (**b**) Mixed EEG with blink contaminated, its **SNR is 0.3970**; (**c**) Independent components of mixed EEG. The first component is eye blink; (**d**) Corrected EEG by our method, its **SNR is 17.6473**.

Figure 8 shows a simulation example which illustrates that the significant change happened in terms of both wave form and SNR after blink correction by our method. Specifically, before eye blink correction, the SNR of mixed EEG was just only 0.3970, and the frontal channels of EEG were significantly contaminated by blinks as shown in Figure 8b. After correction by the proposed method, the blinks were almost totally removed as shown in Figure 8d. Moreover, the SNR of corrected EEG was 17.6473, about 40 times as high as the one before correction.

## 4. Conclusions

In this paper, we raised a premise that the eye blink as an external ICs artifact has a more significant impact on frontal scalp EEG than internal ICs and its power on the front scalp will gradually decay as the distance from the source of eye blink increases. Then a method using correlation index and power distribution is proposed to automatically identify the blink components for scalp EEG data. Additionally, we mathematically proved that the correlation between independent components and scalp EEG channels can be translated directly from the mixing matrix of ICA. It simplified correlation calculating from Pearson product-moment correlation coefficient. Various experimental results confirm the premise proposed in this paper. The method can automatically and directly identify eye blink components without any EOG channels and thresholds after ICA processing. It not only can be applied to the situation where the epoch definitely contains blinks, but also to the situation in which there is no blink in the epoch. Furthermore, the proposed method can be easily used for eye blink artifact removal from a wide range of subjects without setting any threshold parameters or obtaining templates in advance. Therefore, it could be an available approach to detecting blink IC on line. Moreover, the computation of our method is simple, for the correlation index is directly calculated from the mixing matrix ***A*** after ICA. The results of experiments demonstrate that the proposed method jointly using the CBI and power topography characteristics is an automatic way to identify and remove eye blink on the entire dataset without EOG recording.

## Figures and Tables

**Table 1.** The comparison result of four methods on the first dataset with Condition 1.

**Subjects**	**Analysis ICs**	**Correct ICs**				**ICDR**			
	
		**SampEn**	**Template**	**Kurtosis**	**CBI**	**SampEn**	**Template**	**Kurtosis**	**CBI**
1	31	31	31	29	31	100%	100%	93.5%	100%
2	29	27	29	22	29	93.1%	100%	75.9%	100%
3	29	25	26	21	27	86.2%	89.7%	72.4%	93.1%
4	28	28	28	24	28	100%	100%	85.7%	100%
5	36	33	34	19	34	91.7%	94.4%	52.8%	94.4%
**Total**	**153**	**144**	**148**	**115**	***149***	**94.1%**	**96.7%**	**75.2%**	***97.4*%**

**Table 2.** The comparison results of four methods on the second dataset (*θ*—*threshold*).

**Sub**	**Analysis Epochs** **(blinks+no blinks)**	**Correct ICs( Blinks+No Blinks)**				**Accuracy±std(%)**			
	
		**SampEn** **(various *θ*)**	**Template** **(*θ*=0.3)**	**Kurtosis** **(*θ*=1.64)**	**CBI** **(No *θ*)**	**SampEn** **(various *θ*)**	**Template** **(*θ*=0.3)**	**Kurtosis** **(*θ*=1.64)**	**CBI** **(No *θ*)**
1	10+8	8+8	10+7	8+1	9+7	88.9	94.4	50	88.9
2	13+5	7+4	13+5	13+1	12+5	61.1	100	77.8	94.4
3	10+8	6+5	10+7	10+0	9+8	61.1	94.4	55.6	94.4
4	14+4	6+3	14+4	10+0	14+4	50	100	55.6	100
5	18+0	17+0	17+0	18+0	18+0	94.4	94.4	100	100
6	15+3	15+3	14+3	13+0	14+3	100	94.4	72.2	94.4
7	16+2	6+1	15+2	7+0	16+2	38.9	94.4	38.9	100
8	18+0	17+0	18+0	14+0	17+0	94.4	100	77.8	94.4
9	14+4	14+3	14+4	14+0	14+4	94.4	100	77.8	100
10	13+5	11+4	12+5	12+1	13+5	83.3	94.4	72.2	100
total	141+39 =180	107+31 =138	137+37 =174	119+3 =122	136+38 =174	**76.7±21.9**	**96.7±2.9**	**70.1±17.7**	**96.7±3.9**
